# Stingray Venom Proteins: Mechanisms of Action Revealed Using a Novel Network Pharmacology Approach

**DOI:** 10.3390/md20010027

**Published:** 2021-12-24

**Authors:** Kim N. Kirchhoff, André Billion, Christian R. Voolstra, Stephan Kremb, Thomas Wilke, Andreas Vilcinskas

**Affiliations:** 1 Department of Animal Ecology and Systematics, Justus Liebig University Giessen, Heinrich-Buff-Ring 26-32, 35392 Giessen, Germany; Tom.Wilke@allzool.bio.uni-giessen.de; 2 Department of Bioresources, Fraunhofer Institute for Molecular Biology and Applied Ecology, Ohlebergsweg 12, 35392 Giessen, Germany; andre.billion@ime.fraunhofer.de; 3 Department of Biology, University of Konstanz, 78457 Konstanz, Germany; christian.voolstra@uni-konstanz.de; 4 Red Sea Research Center, Division of Biological and Environmental Science and Engineering (BESE), King Abdullah University of Science and Technology (KAUST), Thuwal 23955-6900, Saudi Arabia; stephan.kremb@gmail.com; 5 LOEWE Centre for Translational Biodiversity Genomics (LOEWE-TBG), Senckenberganlage 25, 30325 Frankfurt am Main, Germany; 6 Institute for Insect Biotechnology, Justus Liebig University of Giessen, Heinrich-Buff-Ring 26-32, 35392 Giessen, Germany

**Keywords:** transcriptomics, high-content screening, multi-omics data integration, drug discovery, venomics

## Abstract

Animal venoms offer a valuable source of potent new drug leads, but their mechanisms of action are largely unknown. We therefore developed a novel network pharmacology approach based on multi-omics functional data integration to predict how stingray venom disrupts the physiological systems of target animals. We integrated 10 million transcripts from five stingray venom transcriptomes and 848,640 records from three high-content venom bioactivity datasets into a large functional data network. The network featured 216 signaling pathways, 29 of which were shared and targeted by 70 transcripts and 70 bioactivity hits. The network revealed clusters for single envenomation outcomes, such as pain, cardiotoxicity and hemorrhage. We carried out a detailed analysis of the pain cluster representing a primary envenomation symptom, revealing bibrotoxin and cholecystotoxin-like transcripts encoding pain-inducing candidate proteins in stingray venom. The cluster also suggested that such pain-inducing toxins primarily activate the inositol-3-phosphate receptor cascade, inducing intracellular calcium release. We also found strong evidence for synergistic activity among these candidates, with nerve growth factors cooperating with the most abundant translationally-controlled tumor proteins to activate pain signaling pathways. Our network pharmacology approach, here applied to stingray venom, can be used as a template for drug discovery in neglected venomous species.

## 1. Introduction

More than 200,000 animal species produce venom, mostly for defense and/or predation [1,2,3,4]. Venoms are cocktails of up to 3000 bioactive compounds, including protein/peptide toxins, metabolites and salts [3,5]. Venoms are produced in specialized tissues or glands, and are actively transferred to target organisms via spines, teeth or modified cell-harpoons [3]. Venom targets are found in most major physiological pathways, inducing local effects such as tissue disruption and systemic effects such as paralysis [3,6,7,8]. The diverse components of venoms offer a rich source of novel molecular entities for medical applications. The first example was the cardiovascular agent captopril, which was approved in 1981 for the treatment of hypertension and congestive heart failure [9]. Twelve toxin-based drugs are currently approved for indications such as cardiovascular disease, chronic pain and diabetes, and are derived from species with abundant and/or pure venoms, such as snakes, cone snails, and the gila monster [9,10]. Other toxins are used as research tools to identify new pharmaceutical targets and mechanisms of action (MoA), or for the development of pesticides and cosmetics [11,12]. Animal venoms could help to fill the drug development pipeline because there are hundreds of venomous species each producing up to thousands of bioactive molecules, potentially yielding more than 20 million drug candidates [13].

Animal toxins interact with a vast array of targets [8], but MoAs, toxin synergisms and polypharmacology (the ability to act on multiple targets) have been determined for only a few toxins. The MoA is an elementary property for drug discovery and development [14], which can be used to reduce the side effects often responsible for high attrition rates during clinical trials [15], and to facilitate the development of antivenoms [16]. This knowledge gap must be addressed urgently, given the increasing realization that most drugs act on multiple targets [17,18] and participate in synergistic interactions to modulate complex biological systems [18,19].

**Figure 1:**
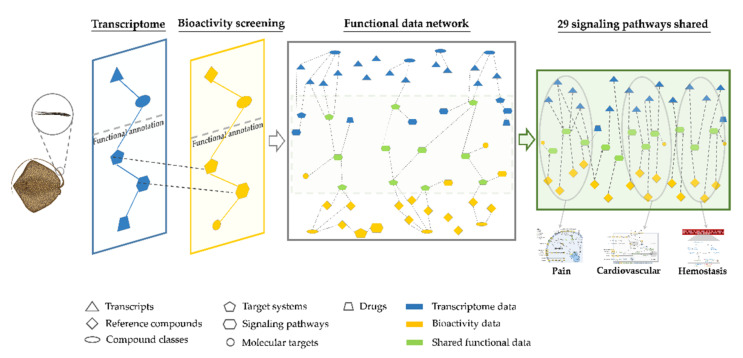
Schematic representation of the integration workflow used to predict the mechanisms of action for drug candidates in crude stingray venom. First, venomous spine tissue from five stingray species was used for transcriptome analysis and high-content bioactivity screening. Second, the functional data inferred from both approaches were integrated, enabling the identification of clusters specific for particular envenomation symptoms that reveal candidate toxins and mechanisms of action. The spine 3D model was reproduced with permission from Dr. Jessica Reichert and the stingray image was based on a photograph taken by Hamid Badar Osmany (www.fishbase.org, accessed on 6 August 2019).

Network pharmacology is a drug discovery tool that integrates multiple omics methods on a functional level to achieve a holistic interpretation of biological responses to specific molecules [20]. Here we report the development of a novel network pharmacology approach for the comprehensive prediction of animal venom protein MoAs (Figure 1). Most venomous species have been neglected as a source of drug leads because their venoms are inaccessible in sufficient quantity and/or purity for analysis, often being extracted as crude venoms contaminated with mucus and the contents of epidermal cells [21]. In particular, many marine, vertebrate, defensive and non-glandular (secretory cells) venoms have been overlooked [21,22,23]. Modern sample-friendly omics approaches can help to overcome these limitations, making venoms more accessible for drug discovery. More than 30% of venomous vertebrates are fish [1], with defensive venom systems (and diverse delivery structures) arising by convergent evolution at least 19 times among 2900 species [24,25]. However, the remote habitats of these species and the typical impurity of venom samples limit our ability to test venom composition and activity. We selected stingrays as a representative system because they best reflect limitations set out above. Stingrays are the most speciose order of venomous cartilaginous fish, including 218 extant marine, brackish and freshwater species featuring defensive tail spines covered with venom-secreting cells, epidermis and mucus [26,27,28]. To better understand the composition and MoAs of stingray venom proteins, we created a functional data network based on integrated gene expression and bioactivity profiles. We analyzed the transcriptomes of venomous spine tissue isolated from five marine and freshwater stingray species to determine which genes were strongly expressed, and thus most likely to encode venom peptides and proteins. We then determined the effect of stingray venom on HeLa cells by image-based high-content bioactivity screening. In the final network pharmacology step, the transcriptomic and bioactivity data were screened against a functional database to create a large data network for comparative analysis, thus increasing the information content. This network combined the original compositional and bioactivity properties with additional target, pathway and druggability data. Specific, curated clusters were extracted from this network to reveal MoAs associated with envenomation symptoms, target systems and pathways. This network pharmacology approach is an inexpensive sample-friendly alternative to traditional methods, providing a comprehensive insight into MoAs and toxin synergisms of crude and neglected animal venoms.

## 2. Results

### 2.1. Crude Venom Transcripts

**Figure A1:**
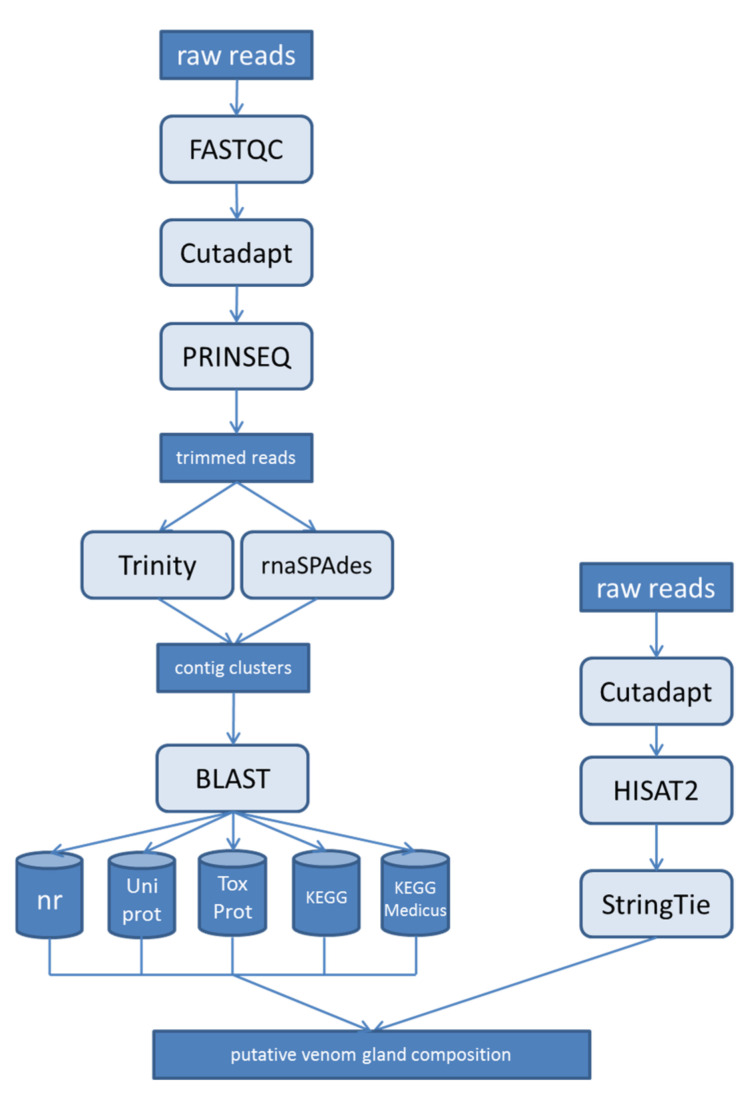
Flowchart showing the bioinformatics workflow used to predict putative venom-gland composition from RNA-Seq reads.

RNA was isolated from five stingray species representing the two most important stingray habitats and the two largest families: Dasyatidae (marine, *Dasyatis pastinaca*, *Himantura leoparda*, and *Pteroplatytrygon violacea*) and Potamotrygonidae (fresh water, *Potamotrygon leopoldi*, and *Potamotrygon motoro*). An average of 30.5 million raw reads were assembled into an average of 2.1 million transcripts per species transcriptome. The bioinformatics workflow is shown in Appendix A (Figure A1), the complete datasets are provided in Datasets S1–S5, quality parameters are listed in Table A1, and the evaluation is summarized in Dataset S9.

**Figure 2:**
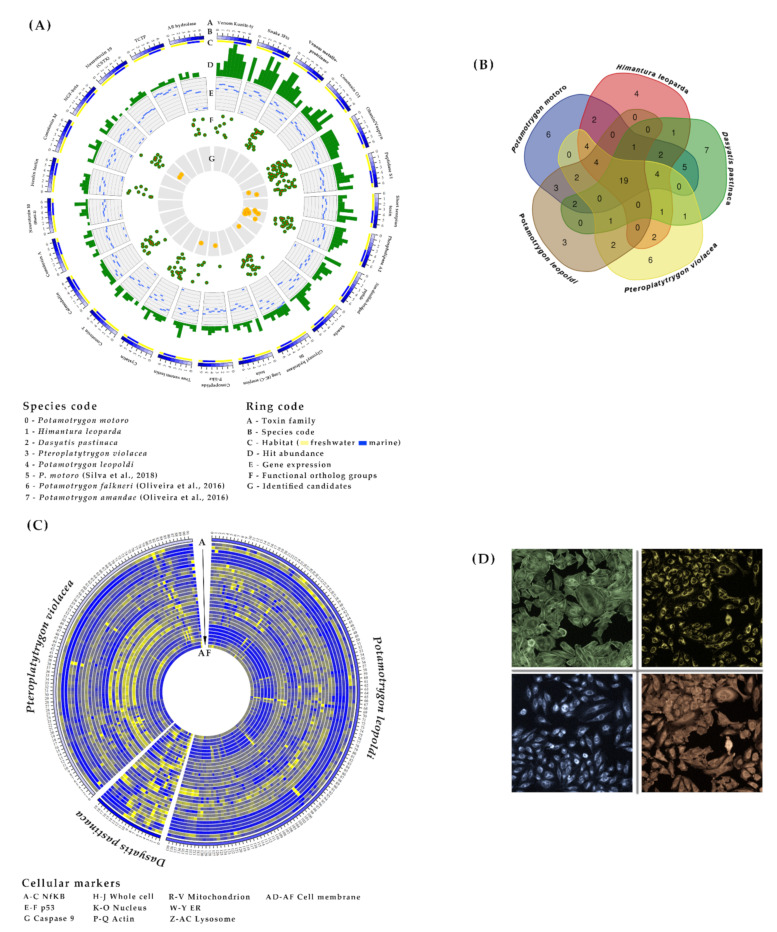
Putative venom composition and venom bioactivity profiles of stingray species. (**A**) The putative venom composition inferred from eight transcriptome datasets (five newly analyzed, and three based on publicly available data denoted in the species code with number 5–7 [29,30]) from seven stingray species, is represented by the top 25 toxin families. (**B**) A Venn diagram based on the toxin family data reveals interspecific similarities. (**C**) The venom bioactivity profile obtained from a high-content screening assay in HeLa cells is represented as a heat map, including 31 highly-informative cytological parameters and 712 reference compounds clustering with stingray venom extracts within a distance threshold of 0.7 (cytological parameters, from outer to inner, are denoted by the letter codes A-AF, which are listed in the Appendix B). (**D**) Images of stained actin (green), ER (blue), Nf-κB (red), and mitochondria (yellow) in HeLa cells.

Transcriptome-inferred crude venom composition and expression patterns should be considered only as a guideline because replicates are missing, and it is difficult to distinguish between toxins and physiological proteins. Even so, venom composition based exclusively on toxin-related annotations against the ToxProt database indicated high interspecific similarity, with 24% of toxin families overlapping in all five stingray transcriptomes, representing 65–74% of all hits (Figure 2A and Dataset S9). This is translated to 19 shared toxin families, among which the translationally-controlled tumor protein (TCTP) family was the most strongly expressed in each species, often directly followed by the glycosyl hydrolase 56 (hyaluronidase) family, although hyaluronidase expression was low in *H. leoparda* and *P. violacea*. The shared family dataset also included the ohanin/vespryn and phospholipase A2 (PLA2) families, and the widely distributed venom kunitz-type, venom metalloproteinase (M12B), and peptidase S1 toxin families (Dataset S9). Members of the calmodulin family and several putative neurotoxins representing the snake three-finger toxin (TFT) family were also strongly expressed in stingray venom tissue. However, some toxin-like transcripts matched proteins/peptides with no family attribution such as the augerpeptide hhe53, which was found in all five transcriptomes and was the most abundant transcript in *H. leoparda*, *P. leopoldi* and *P. violacea*. From a biochemical perspective, the stingray venom mainly featured enzymes and other proteins, but has just a low content of peptides (<50 amino acids), representing only ~6% of the annotated hits against ToxProt when fragments were ignored (Dataset S9).

We also conducted an interfamilial comparison of the crude venom transcripts between the marine (Dasyatidae) and freshwater (Potamotrygonidae) species (Figure 2B). Differences were only considered when more than one species shared the same pattern. The most remarkable difference was the stronger expression of the true venom lectin family in marine species, contrasting with the low or absent expression in freshwater species.

### 2.2. Venom Bioactivity

Crude venom from three stingray species (*D. pastinaca*, *P. violacea*, and *P. leopoldi*) was tested in a high-content screening (HCS) assay based on HeLa cells. The species were selected based on their comparability in the transcriptome dataset and the availability of sufficient fresh venom samples for bioactivity screenings. The raw data were reduced in a stepwise manner to 31 highly informative cytological parameters (Appendix B). The raw and reduced datasets, as well as lists of reference drugs (clustering with our crude venom extracts), are listed in the Datasets S9 and S10.

The cytological profiles of all three stingray venom extracts revealed dose-dependent effects against the NF-κB pathway and the cytoskeleton, as well as the nucleus, mitochondria, endoplasmic reticulum (ER) and lysosomes (Figure 2C,D). These results indicated a slight increase in the translocation of NF-κB into the nucleus, the degradation of F-actin, nuclear chromatin condensation, impairment of mitochondrial thiol-related pathways, a loss of ER integrity, and the depletion of lysosomes. In all cases the effects were strongest for the undiluted venom extract of *P. violacea*. The cytological profiles of stingray venom clustered with those of ~29% of all the reference compounds we tested. This revealed that drugs with similar bioactivity profiles to crude stingray venoms (and probably similar MoA) mainly target the nervous and cardiovascular systems, but also the immune system (e.g., anti-inflammatory and anti-allergic drugs).

The comparison of marine and freshwater species revealed that marine but not freshwater stingray venom induces effects against the ER and lysosomes. In addition, *P. violacea* venom triggered the translocation of p53 into the nucleus, membrane disruption (resulting in the cytoplasmic redistribution of the membrane marker), ~40% cell death, cell cycle arrest during the G0/G1 phase, and a strong increase in the number of cells with a low DNA content, indicating damaged (necrotic) or apoptotic cells. Interestingly, 11 of 12 reference compounds clustering with the (marine) *D. pastinaca* venom profile overlapped with those clustering with (marine) *P. violacea* venom but none of them overlapped with (freshwater) *P. leopoldi* venom.

### 2.3. Network Pharmacology

The transcriptome and bioactivity datasets each offer a limited perspective on the MoA of stingray toxins. However, the integration of both data topologies at the same functional level allows a more holistic interpretation. Therefore, stingray toxin-like transcripts and reference compounds with similar MoAs to our stingray venom extracts were screened against the KEGG database, linking the transcripts and clustered drugs to molecules with known targets, target systems, pathways and associated diseases. An average of 32% of all stingray transcripts and 58% of clustered reference compounds were annotated in this manner. The datasets were integrated at a pathway level because this was the most informative and abundantly inferred functional category. The pathways inferred by transcript and reference compound annotation were compared, resolving the integrated dataset to a total of 216 pathways among which 29 were shared and represented 15 target systems, including the *Nervous system*, *Circulatory system*, *Cancer*, *Signal Transduction*, and the *Immune system*. We focused on this shared dataset, which included 70 transcripts representing 16 toxin families and 70 reference drugs clustered by HCS mostly neuropsychiatric and cardiovascular agents. The core dataset included the interlaced toxin-like transcripts, cellular bioactivities, and functional data enabling the prediction of stingray toxins MoAs against individual target systems and pathways in the context of specific envenomation outcomes.

**Figure 3:**
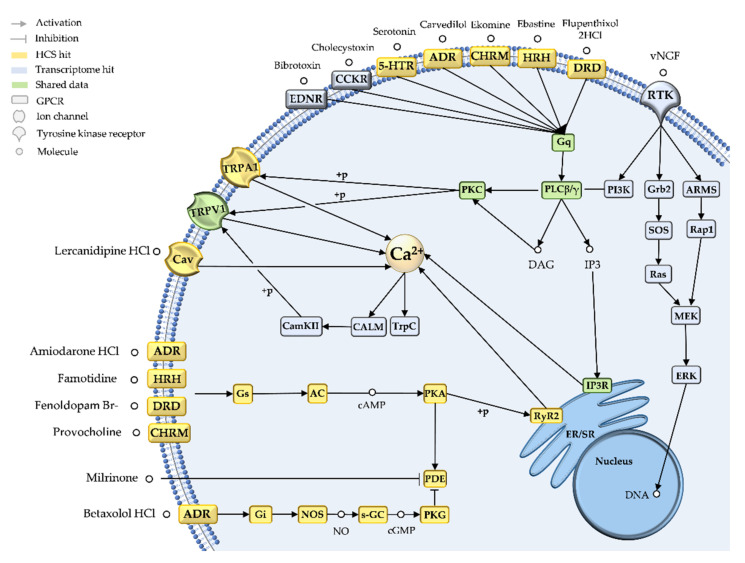
Pain cluster based on integrated transcriptomic and bioactivity data. The integration of transcriptomic (blue icons) and high-content bioactivity screening data (yellow icons) predicts the mechanism of action of pain-related drug candidates in stingray crude venom. Green icons show consistencies in both datasets.

Fish venom triggers severe pain as the primary envenomation symptom, and we congruently identified nine pathways in the core dataset with potential roles in pain. These pathways represented the *Signal transduction*, *Nervous system* and *Sensory system* categories and were targeted by 20 transcripts and 41 HCS-clustered reference drugs (Appendix C). The manual curation and graphical representation of molecular targets and associated signaling cascades in the pain-related pathways allowed us to predict the MoA of pain-inducing stingray venom components in the nervous system, which we refer to hereafter as the pain cluster (Figure 3).

Our pain cluster clearly indicated that G-protein coupled receptor (GPCR) calcium signaling is a key MoA for pain-inducing stingray toxins. Specifically, the transcriptome hits bibrotoxin and cholecystoxin, and 16 of the HCS hits (including ebastine, ekomine, and carvedilol) were found to target distinct GPCRs (5-HTR2, HRH1, ENDR, CC2KR, or ADR), subsequently activating a Gα protein (Gαq). This in turn causes phospholipase C (PLC) to hydrolyze a membrane substrate into inositol-1,4,5-triphosphate (IP3) and diacylglycerol (DAG). IP3 acts as a second messenger, binding to its receptor in the ER or sarcoplasmic reticulum (SR) and triggering intracellular calcium release. DAG activates protein kinase C (PKC), which phosphorylates targets such as TRPV1, a non-selective membrane cation channel, contributing to further intracellular calcium accumulation.

The activation of calcium signaling was supported by the remaining pain cluster hits, albeit acting via different signaling pathways. Three hits in the transcriptome dataset represented venom nerve growth factor 1 (vNGF), which binds a receptor tyrosine kinase (RTK) and directly activates either PLC or PKC (Figure 3). NGFs can activate extracellular signal-regulated kinase (ERK), a transcriptional regulator with key roles in several pain states [31]. Nine additional HCS hits activate protein kinase A (PKA) via GPCRs, and subsequently release calcium from the ER via the ryanodine receptor RyR2 (Figure 3). Intracellular calcium modulation was also supported by HCS hits on voltage-gated calcium channels and TRPA1, a non-selective sodium/calcium channel. However, six HCS hits provided evidence for the depletion of intracellular calcium, although the subsequent calcium-modulated reactions include the induction of metabolic processes, muscle contraction, exocytosis, autophagy, apoptosis, neurogenesis or neuronal transmission [32,33]. EF-hand proteins are involved in several of these processes, and our transcriptome dataset included 11 additional hits for the calmodulin homolog calglandulin.

**Figure A2:**
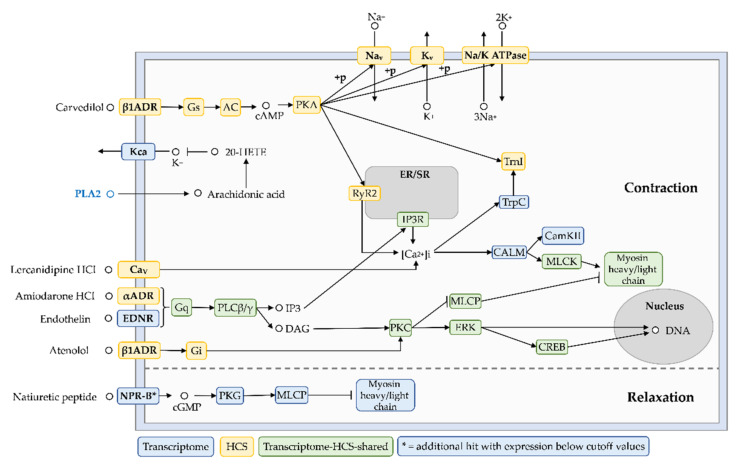
Cardiovascular cluster. Twelve pathways in the integrated transcriptome and high-content screening dataset reveal the presence of 18 putative cardiotoxins, which together with 14 clustered drugs can be used to predict the target modulation by this toxin class and the cellular response. This cluster indicates alternating vascular and/or cardiac muscle contraction and relaxation periods, for example based on MLCP inhibition or activation and Na^+^ and K^+^ mobilization.

**Figure A3:**
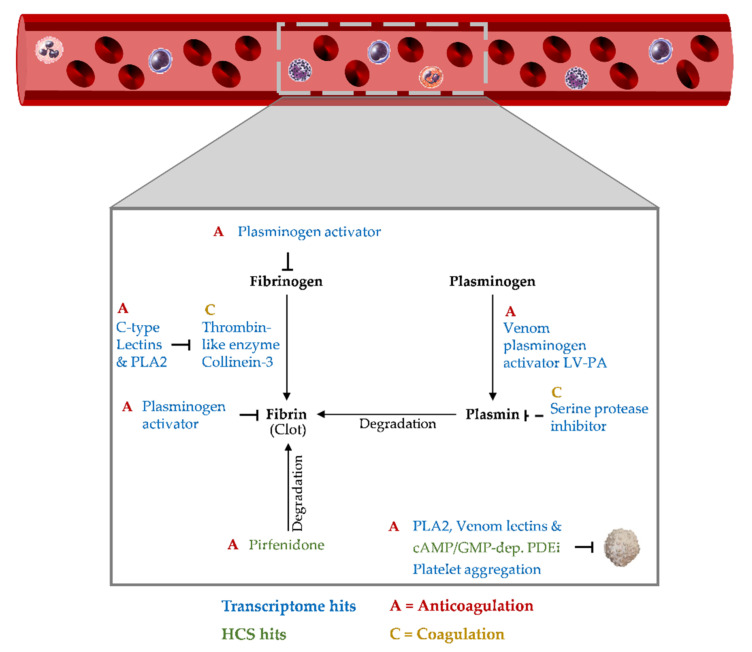
Hemostasis cluster. Twelve pathways in the integrated transcriptome and high-content screening dataset reveal the presence of nine putative hemotoxins which together with 14 clustered drugs and the corresponding functional data give insight into their potential mechanisms of action. This cluster strongly suggests that anticoagulants, especially those with fibrin(ogen)lytic activity, play a key role.

Our novel network pharmacology approach has a remarkable multiplexing capacity. Depending on the focus, multiple clusters can be built from our integrated shared core functional network, including cardiovascular and hemostasis clusters (Appendix C, Figure A2 and Figure A3). Briefly, the cardiovascular cluster included 14 HCS reference drugs and 17 transcriptome hits against putative cardiotoxins: one endothelin, one cholecystokinin, 15 (acidic/basic) PLA2 homologs, and a natriuretic peptide as another candidate although it was below our expression level cutoff. The highlighted MoA in this cluster indicated the activation of vascular and probably cardiac contraction and relaxation phases via the PKA, PKC, or PKG pathways, which subsequently activate or inhibit myosin heavy and light chains. In the hemostasis cluster (Figure A3), we identified nine hemotoxins with potential fibrin(ogen)olytic activity, including two venom lectins, a serine proteinase inhibitor, a venom plasminogen, a thrombin-like enzyme, and four (acidic/basic) PLA2 homologs.

## 3. Discussion

Animal venoms are a rich source of potent and diverse new drug candidates that could provide large insights into novel MoAs and synergistic activities.

Stingray venom is typically characterized by gel electrophoresis, zymography, in vivo assays or mass spectrometry, usually focusing on freshwater species from the genus *Potamotrygon* [26,34,35]. Transcriptome analysis has also been carried out for three *Potamotrygon* and one *Neotrygon* species [29,30,36]. These studies reported the presence of enzymes, but also other proteins and peptides in the mass range 1–276 kDa. Stingray venom is transferred via a serrated tail spine, causing severe injuries and typical fish envenomation symptoms: intense pain, hemorrhage, edema, erythema, hypotension, secondary necrosis, and infection [34,37]. Our transcriptome and HCS data confirmed the general compositional and functional properties discussed above, including apparent inflammatory, tissue and hemostasis disrupting activity.

The high abundance of transcripts representing the TCTP and hyaluronidase families in our transcriptome datasets agrees with previous findings in *Potamotrygon* species and is commensurate with the symptoms of stingray envenomation [33,34]. TCTP toxins are thought to induce edema [38], whereas hyaluronidases act as venom spreading factors and have been found in two *Potamotrygon* species [26,39]. Interestingly, the putative hyaluronidases we identified were expressed at high levels in both marine and freshwater species, contrasting with the absence of hyaluronidase activity in marine stingray *Dasyatis guttata* [26] and hyaluronidase sequences in the *Neotrygon kuhlii* venom proteome [36].

**Figure A4:**
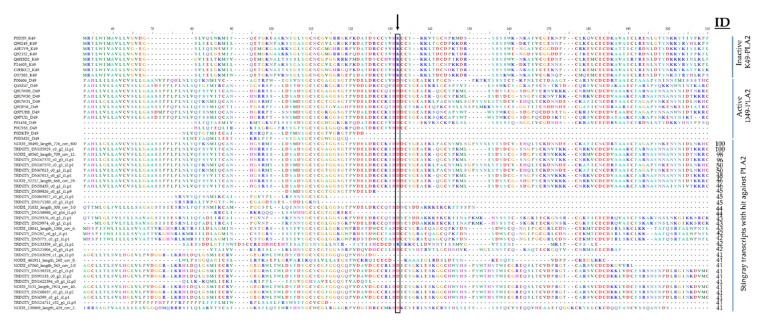
Alignment of stingray venom transcripts attributed to the PLA2 toxin family and reference sequences of the active D49 and inactive K49 PLA2 subfamilies from UniProt. The arrow and box show position 49 of the mature protein. All the stingray transcripts we identified belong to the active D49 PLA2 subfamily.

Stingray venom modulates hemostasis by delaying coagulation via the activity of fibrin(ogen)olytic enzymes [40]. Accordingly, we identified serine proteases and metalloproteinases in all our transcriptome datasets, supporting the bioactivity data and providing the basis for the hemostasis cluster (Figure A2). The abundance of PLA2 transcripts also agrees with the reanalysis (for comparability) of the *Potamotrygon* transcriptomes reported previously (Datasets S6–S8) [29,30] and corresponding enzymes have been identified in nearly all animal venoms analyzed thus far. However, only minimal PLA2 activity was found in stingray venom in one previous study [26], and another study claimed that PLA2 has never been detected in fish venom [21]. A possible explanation for this discrepancy is the presence of catalytically inactive K49-PLA2 isoforms as found in snake venoms, which would generate transcriptome hits even in the absence of activity [41]. However, our transcriptome hits solely indicated the presence of active D49-PLA2 (Appendix D, Figure A4) and the role of this enzyme family therefore requires further investigation.

**Figure A5:**
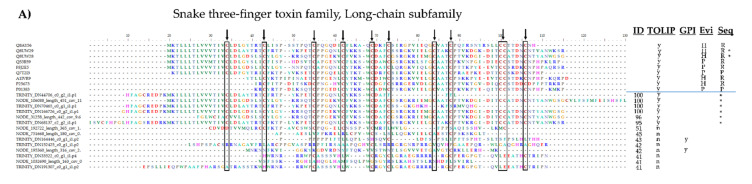

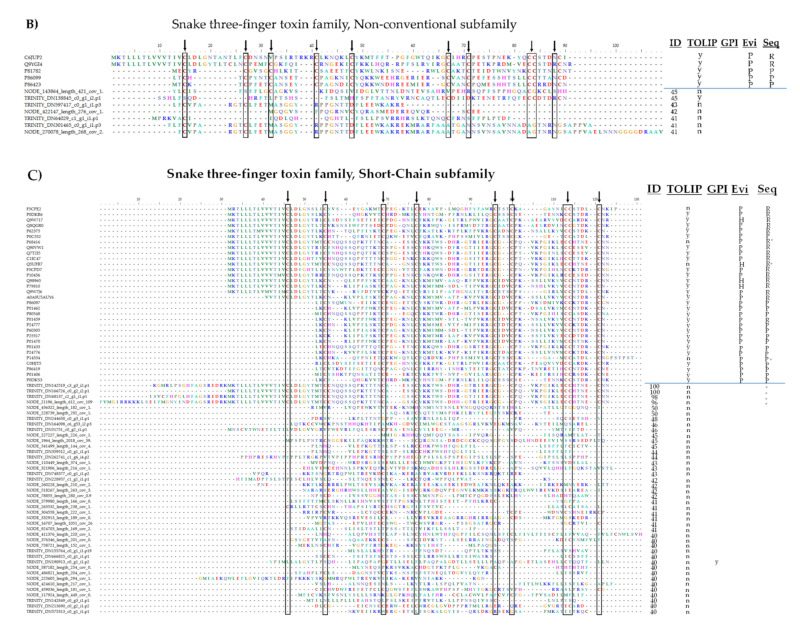
Alignment of stingray venom transcripts attributed to the Three-finger toxin family (TFT) and their corresponding hits from Uniprot separated by (**A**) Long-chain, (**B**) Non-conventional, and (**C**) Short-chain subfamilies. The arrow and box denote the characteristic TFT cysteine scaffold leading to the ‘three-finger’ fold. The table attached to the alignments informs on the sequence identities of stingray transcripts to the databased TFT, the presence (y) or absence (n) of the TOLIP domain attributed to TFT only, the presence (y) of a GPI anchor known from non-toxic three finger proteins, evidences on protein level (P) or only by homology (H), and if the sequence database is a translated mRNA (R) or a ‘real’ protein sequence (P). The asterisk (*) links the best hits of stingray transcripts with their annotated TFT.

Our transcriptome analysis revealed several intriguing results. First, the augerpeptide was present in all of our transcriptome datasets and was often the most abundant transcript, supporting its detection in the first *P. motoro* transcriptome study [30]. However, the activity of this peptide remains unknown. Second, calglandulin was identified in our transcriptome datasets and in three previously reported transcriptomes representing the genus *Potamotrygon* [29,30], but calglandulin was previously suggested to be a component of the venom secretion process rather than the venom itself [42]. However, the importance of calcium signaling during envenomation and the activation of EF-hand proteins by calcium may indicate that calglandulin has additional roles in the venom that require further investigation. Finally, we identified abundant, high-ranking transcripts representing several neurotoxins of the snake TFT family in our five stingray transcriptomes and in the reanalysis of existing *Potamotrygon* transcriptome data [29,30], supporting the presence of this family even if the expression level is typically low (Datasets S6–S9). For further clarification of these hits, we assessed our transcripts for the characteristic TFT patterns (i.e., cysteine scaffold, TOLIP domain, and the absence of certain post-translational modifications) and found that at least 10 transcripts fulfilled all criteria and thus confirm the presence of TFT-like proteins in stingray venom (Appendix D, Figure A5). Despite a number of studies indicating a stronger relationship between the transcriptome and proteome, in terms of presence/absence if not abundance [43], the augerpeptide, calglandulin and TFT hits, as well as the PLA2 transcripts and the marine hyaluronidases, require further confirmation at the protein level.

Our bioactivity assay revealed compartmental signals that provide insight into the cellular responses to stingray venom. Although the precise MoAs remain unknown, the translocation of Nf-κB, mitochondrial impairment, and the disruption of the ER and lysosomes indicate oxidative stress at the cellular level [44]. The mitochondrial signal in particular matches previous finding that venom of the marine stingray *Pastinachus* (*Dasyatis*) *sephen* alters mitochondrial membrane potential by enhancing the production of reactive oxygen species [45]. In the ER and lysosome, swelling, loss of membrane integrity, and even toxin accumulation can contribute to the observed effects [44,46,47,48]. Another scenario for the loss of organelle and plasma membrane integrity is the observed degradation of the cytoskeleton triggered by *P. violacea* venom, as also reported for cnidarian toxins [49]. The loss of plasma membrane integrity may explain the cytotoxicity of marine *P. violacea* crude venom in our bioactivity assay. Contradictorily, it appears that the *Potamotrygon* crude venom did not induce cell death on our bioassay, although necrosis in freshwater envenomations is typically remarkably severe [26,34]. Given the inferred importance of calcium signaling as a target of stingray toxins, the disruption of the ER, mitochondria, and lysosomes may reflect their role as intracellular calcium stores [33].

Each dataset provided only limited insight into the MoAs and synergistic activity of stingray venom components, so to achieve a more holistic interpretation we integrated the gene expression and bioactivity data in a novel network pharmacology approach. Several fields, including natural product research, drug discovery, and pharmacology, typically involve large-scale screens based on target binding or MoAs [50], but more recent trends combine these approaches to provide data on compound-target interaction as well as on-target and off-target MoAs [51,52]. Special emphasis is placed on the identification of key nodes that modulate system phenotypes in the context of multi-target drugs and drug combinations [18]. Our approach adds to the growing number of studies addressing this knowledge gap. The resulting data network revealed that the functional integration of omics data can identify MoAs and potential synergistic interactions for multiple system perturbations visualized as clusters for major stingray envenomation symptoms.

The primary effect induced by stingray venom is pain. The induction or modulation of peripheral pain by animal toxins is often linked to ligand-gated or voltage-gated ion channels, such as TRPV1 and Nav1.7, respectively [53]. However, our pain cluster suggested that the major pain-inducing mechanism of stingray toxins is the activation of calcium signaling via GPCRs. Three major GPCR-dependent pain pathways are known—the excitatory IP3 and PKA pathways, and the inhibitory PKG pathway [54]—and all three were highlighted by our pain cluster. However, the main route synergistically activated by two stingray venom transcripts and supported by 16 HCS hits appears to be the GPCR-IP3 pathway. Several lines of evidence indicate that GPCRs in nociceptors (pain-perception neurons) are targeted by venom proteins, especially those from marine animals [54]. For example, sea anemone gigantoxin I activates the GPCR-IP3 cascade, followed by the phosphorylation of TRPV1 and calcium release [55]. Stonefish verrucotoxin appears to act as a β1-adrenoceptor agonist, increasing L-type calcium currents via the PKA pathway and inducing neurotransmitter release [56]. Furthermore, the synthetic ω-conotoxin ziconotide (an approved drug) and two α-conotoxins from the cone snail block N-type calcium channels in response to inhibitory GPCR signaling [57,58].

The perception of severe pain may involve the combined activation of several pathways [59]. In our pain cluster, we identified multiple targets that activate calcium signaling via different routes (IP3, PKA, and also by directly acting on ion channels). However, we also found evidence for alternative routes, such as NGFs that induce pain via RTK signaling and downstream ERK and IP3 pathway activation [31]. This is often related to inflammation, another well-documented stingray envenomation symptom. The multiple predictions of serotonin and histamine receptor activation in our pain cluster support the intimate relationship between inflammatory mediators and nociceptor hypersensitivity, sometimes leading to hyperalgesia [59]. Nociceptors can be activated directly by venom-related histamine, bradykinin or serotonin, as previously suggested for stingray venoms, or toxins mimicking their activity [60,61]. However, indirect activation is also possible when venom compounds induce the massive release of endogenous inflammatory mediators, for example by mast cell degranulation [60]. The abundant TCTP transcripts in our datasets may encode key mediators of this process by strongly inducing the release of histamine [38], although the precise mechanism is unknown. The synergistic activity of TCTPs and hyaluronidases may further trigger the severe necrosis often associated with stingray envenomation [34]. Necrosis is often more severe following envenomation by freshwater stingrays, and we congruently identified nine HCS hits for HRH1 that were exclusively related to the bioactivity profile of *P. leopoldi*.

We also found strong evidence for the polypharmacology of stingray venom components. The candidate pain-inducing toxins in our pain cluster were bibrotoxin and cholecystoxin, which are associated with neuropathic pain and inflammation but also cardiovascular disorders [62,63]. Accordingly, these candidates were not only identified in the pain cluster, but also in the cardiovascular cluster (Figure A2). Their activity in each cluster may involve different second messengers, but ultimately cause the release of intracellular calcium thus matching our prediction of IP3-calcium pathway activation.

Taken together, our results agree with previous network pharmacology data showing that multi-omics data integration can provide robust predictions of MoAs and synergies in animal venoms that have not been characterized in detail. Our transcriptome and bioactivity data indicate that stingray venom includes enzymes, other proteins and a small number of peptides that can induce pain, disrupt the tissue matrix and hemostasis, and induce pro-inflammatory and cardiotoxic activity. Our integrative approach relies on the quality and abundance of functional data as well as additional manual curation. However, the integrated data network and resulting clusters comprehensively unraveled the mechanisms underlying major stingray envenomation symptoms and their timeline. First, pain is mainly induced via GPCR-IP3 signaling and potentiated by GPCR-PKA, NGF-ERK/IP3, and voltage-gated ion channel activation. Second, inflammation triggers further pain based on the massive release of intracellular histamine, for example by strongly expressed TCTP toxins. Finally, tissue disruption leads to the typical necrotic profile probably reflecting the combination of strongly expressed hyaluronidases and massive inflammation. Our network pharmacology approach was therefore able to identify several routes via which stingray venom synergistically induces system perturbations, but we also found initial evidence for the polypharmacological nature of stingray toxins that trigger multiple systems such as nociception, cardiovascular and immune.

## 4. Materials and Methods

### 4.1. Crude Venom Extracts and cDNA Library Preparation

Venomous tissue (45–212 mg) was scraped from the tail spine of mature live specimens of two freshwater stingrays (*P. leopoldi* and *P. motoro*; Potamotrygonidae) and three marine stingrays (*D. pastinaca*, *H. leoparda* and *P. violacea*; Dasyatidae) following Directive 2010/63/EU on the protection of animals used for scientific purposes. Total RNA was extracted for mRNA isolation, cDNA library construction and sequencing (50 million paired-end reads) carried out by Vertis Biotechnologie (Freising, Germany) and Macrogen (Amsterdam, Netherlands) using the Ilumina HiSeq2000 platform. For high-content bioactivity screening, crude venom was obtained from 30–104 mg of spine tissue from mature live specimens of *P. leopoldi*, *D. pastinaca* and *P. violacea* using a standardized methanol-based bioactive compound extraction protocol (Appendix B, Table A2).

### 4.2. Venomous Tissue Transcriptome Analysis

The transcriptome analysis workflow is shown in Figure A1. Briefly, 182 Gb of sequence data from five new stingray transcriptomes (species listed above, one specimen per transcriptome) and three existing datasets [29,30] were quality checked, trimmed and assembled using a combination of Trinity [64] and rna-SPAdes [65] to reduce algorithm-specific peculiarities (Table A1). Gene expression levels were calculated by mapping raw reads back onto the assembled transcripts using HISAT2 [66] and sequences with low coverage (TPM < 0.5) were discarded. Translated transcripts were annotated by sequence comparison in BLAST [67] and HMMER [68] using UniProt [69] and Pfam [70] as references, followed by venom-related annotation solely against ToxProt [71]. Annotations were filtered by identity (≥40%), coverage (≥40%), and bit score (≥30) and only the highest-scoring hit of the remaining transcripts against a unique UniProt ID was taken into account. Putative toxin family classification was based on ToxProt and was collapsed at the superfamily level where possible. Toxin family expression is presented as relative values (the sum of the expression values of single hits in a single toxin family divided by the number of these hits). Transcripts were further annotated against KEGG [72] and KEGG Medicus [73] to improve cross-linking to the bioactivity data and to identify signaling pathways and target systems. Functional category names from KEGG are herein denoted with capital and italics.

### 4.3. High-Content Bioactivity Screening (HCS)

We compared the effects of stingray venom extracts to the effects induced by 712 reference compounds from the LOPAC 1280 library (Sigma-Aldrich, Steinheim, Germany) in a HeLa cell bioactivity assay. Briefly, serially-diluted venom extracts were tested in four replicates. After 24 h, four fluorescence-based cell-staining protocols (Appendix B) were used to stain nine cellular targets (Table A3). HCS was carried out on the Cellomics ArrayScan VTI platform (Thermo Fisher Scientific, Waltham, MA, USA) equipped with a 10x objective EC Plan Neofluar (Zeiss, Oberkochen, Germany). Images were analyzed using the Compartmental Analysis Bio Application (Cellomics). At least 500 valid objects were analyzed per well. Cell loss and cycle analysis was carried out in parallel using the Cell Cycle Bio Application (Cellomics) and a minimum of 2000 valid objects. Raw data were processed for stepwise data reduction and statistical evaluation until a final set of 31 highly informative parameters remained.

For each reference compound and crude venom sample, the induced cell responses were aggregated into high-resolution cytological profiles, defined as unique fingerprints of cellular perturbations generated by joining distinct features derived from spatially-resolved measures of fluorescence intensities relative to control treatments [74]. These profiles were processed by hierarchical clustering (complete linkage clustering and Spearman rank correlation) in Multi Experiment Viewer v4.9 [75]. Clusters were defined as compounds (venom-associated or references) that fell within a distance threshold of 0.7. We assumed that similarities in phenotypic responses among compounds indicate similar biological targets and MoAs [52,76]. Therefore, as described above for transcriptome analysis, reference compounds clustering with crude venom profiles were manually annotated against KEGG, linking the phenotypic responses to the transcriptome-based activity profile.

### 4.4. Integrative Approach

The functional data inferred independently by transcriptome analysis and bioactivity screening were integrated to create a dataset of shared signaling pathways including functionally annotated hits generated by both approaches. From this integrated dataset, single clusters were identified for each envenomation effect, as herein, but can also be identified for target systems and toxin families/classes. The cluster for the primary stingray envenomation effect (pain) was created by combining all pathways potentially related to this effect and using the hits in these pathways to reveal potential molecular targets and induced cellular responses either by functional annotation in KEGG or additional manual curation. Finally, the venom transcripts, molecular targets and cellular response data were compiled in specific clusters to identify drug candidates in the stingray venom and predict their major MoAs.

## Data Availability

Transcriptome datasets analyzed in this study are publicly available and can be found here: https://www.ncbi.nlm.nih.gov/sra under accession numbers SRR1104920(4)-8 (BioProject number: PRJNA605588), SRR2039259, and SRR7994305, and https://db.cngb.org under accession number CNP0000235 (the latter three transcriptomes were last accessed on 8 May 2019) The raw high-content bioactivity data presented in this study are available in Dataset S10.

## Supplementary Materials

The following are available online at https://www.mdpi.com/article/10.3390/md20010027/s1, Datasets S1–S10.

## Appendix A. De Novo Transcriptome Assembly, Expression, Annotation, and Functional Pre-Integration

The transcriptome analysis workflow was developed to predict the putative venom-gland composition from RNA-Seq reads (Figure A1 and Table A1). Raw reads were initially reviewed using FastQC v0.11.8 [77] following adapter trimming using cutadapt v1.16 [78]. Next, all read ends were trimmed according to their quality score using a window size of 1 and reads with an average quality score < 20 were discarded using PRINSEQ v0.20.4 [79]. Assembly was carried out using a combination of Trinity v2.8.4 [64] and rnaSPAdes v3.13.0 [65] to reduce algorithm-specific peculiarities. Trinity was used with default parameters plus modifications for the reconstruction of small peptides by applying min_contig_length = 60 as well as trimomatic (SLIDINGWINDOW:4:5, LEADING:5, TRAILING:5, MINLEN:25), cpu (128) and max_memory = 1200 G. We also used rnaSPAdes with default parameters plus modified values for cpu (128) and memory (1200) to increase the calculation speed. Both reconstructions were compared by BLAST and 100% identical contigs (identity = 100%, query_coverage = 100%, subject_coverage = 100%) were removed. The resulting dataset was translated using TransDecoder v5.1.0 [80] to extract open reading frames with a minimum amino acid length of 20, allowing the recovery of peptides and even toxin fragments.

**Table A1:** Summary statistics for the stepwise quality control and annotation of the five transcriptomes in the present study and the three existing and reanalyzed potamotrygonid transcriptomes.

	*Potamotrygon* *motoro*	*Potamotrygon leopoldi*	*Himantura* *leoparda*	*Dasyatis* *pastinaca*	*Pteroplaty* *-trygon* *violacea*	*Potamotrygon* *motoro ^a^*	*Potamotrygon* *falkneri ^b^*	*Potamotrygon* *amandae ^c^*
**Raw reads**	33.218.150	27.144.363	30.544.874	29.866.375	31.603.727	38.674.474	7.687.715	7.269.571
**Length**	150	100	150	150	100	90	35-252	35-252
**Assembled contigs**	1.920.972	2.008.981	2.229.479	697.969	3.729.178	2.545.786	306.784	562.924
**Protein sequences** **Transdecoder**	13.813.790	4.512.397	5.886.854	2.002.545	7.811.707	10.054.481	2.537.742	4.496.691
**Annotation contigs in UniProt**	37.583	22.400	36.133	28.976	22.064			
**Annotated contifs in UniProt (with expression > 0)**	18.034	8157	16.859	28.976	6878			
**Unique UniProt hits**	7120	3660	6673	9949	2713			
**Annotated contigs in ToxProt (unfiltered)**	899.886	435.270	565.171	179.645	776.647	975.812	1.047.614	407.053
**Total ToxProt hits** **(filtered by expression >0, ID, coverage, bit-score)**	776	214	1229	499	193	388	403	435
**Unique ToxProt hits** **(filtered by expression >0, ID, coverage, bit-score)**	147	84	160	122	129	154	78	97
**Unique annotated Toxin Families (collapsed on superfamily level where possible)**	53	38	43	44	46	57	33	41
**Annotated contigs in KEGG**	56	24	43	63	22	64	52	57
**Annotated unique KEGG Ortholog Group**	19	12	24	26	9	31	24	32
**Annotated KEGG Pathway**	72	68	69	68	43	90	68	80
**Annotated KEGG Drugs**	4	3	1	12	1	4	2	4
**Pathway overlap with HCS**	22	22	26	27	16	58	24	26

^a^ Reanalyzed transcriptome of *Potamotrygon motoro* [30]. ^b^ Reanalyzed transcriptome of *Potamotrygon falkneri* [29]. ^c^ Reanalyzed transcriptome of *Potamotrygon amandae* [29].

For annotation, sequences were compared to NCBI-NR (release-2019_03), UniProt (release-2019_05), ToxProt (release-2019_05), KEGG (release-2018_12) and KEGG-Medicus (release-2019_03) using BLAST v2.8.1 [81] to identify homologous sequences and transfer putative functionalities. All results were initially filtered by identity ≥ 40%, query_coverage ≥ 40%, subject_coverage ≥ 40% and score ≥ 30. Multiple hits against a single reference protein were collapsed by only using the hit with the highest score. Domains were annotated using HMMER3 v3.2 [82] with PfamA (release-2019_03) models and default parameters. Results were filtered by E-value (<1 × 10^−17^) and coverage (>0.45).

To quantify the expression of transcripts, adapter-trimmed raw reads without further quality trimming were mapped using HISAT2 v2.1.0 [66] against the reconstructed transcriptome to detect most possible transcription variants. Abundance was calculated using StringTie v1.3.6 [83] with a minimum length of 60 and a minimum read coverage of 2 allowed for the predicted transcripts. Abundances are displayed in TPM (transcripts per million, normalized expression level in RPM units) to allow indirect comparison between samples. Due to the nature of the probes, no biological replicates were available and therefore a valid differential gene expression calculation was not possible.

## Appendix B. Experimental Details and Data Analysis of High-Content Screening

**Table A2:** Sampled species, tissue amount, extraction method, and methanol volume for extraction of stingray crude venoms for the high-content bioactivity screening assay.

Species	Dry Weight (mg)	Extraction Method	Extraction Volume (µL)
*Potamotrygon leopoldi*	35	Methanol	500
*Dasyatis pastinaca*	30	Methanol	500
*Pteroplaytrygon violacea*	104	Methanol	1000

Tissue extraction protocol. Tissue was frozen in liquid nitrogen and powdered with a mortar (Table A2). The powder was dissolved in 1 mL 98% methanol per 100 mg tissue, mixed, and incubated at 30 °C for 1.5 h. After overnight incubation at 4 °C, the mixed samples were heated to 30 °C, centrifuged and the supernatant divided into aliquots of 100 µL and lyophilized.

Assay preparation. A dilution series was prepared by reconstituting the lyophilized aliquots in 500 µL Dulbecco’s modified Eagle’s medium (DMEM). HeLa cells were seeded into 384-well plates at a density of 2000 cells per well in 25 μL DMEM and were incubated for 24 h at 37 °C in a 5% CO_2_ atmosphere. The cells were treated with 25 μL of the re-dissolved samples or reference compounds in four replicates for 24 h.

Staining protocols. Cell staining was carried out as previously described [84]. After fixation in 4% formaldehyde for 20 min, cells were permeabilized, blocked, stained, and/or repetitively washed using the Cellomics HCS reagents (Thermo Fisher Scientific): Wash Buffer (WB), Wash Buffer II (WBII), Blocking Buffer (BB) and Permeabilization Buffer (PB). Following the last staining step, all plates were washed three times with WB, sealed, and stored at 4 °C for imaging. The nine cell compartments were categorized and stained using four different staining protocols (panels) as described below.

Panel 1 (actin): Fixed cells were permeabilized for 15 min and blocked for 15 min before adding 12.5 μL of the primary staining solution (cell culture medium) containing 3.6 μL/mL phalloidin-FITC (0.5 mg/mL in methanol; Cat. P5282, Sigma Aldrich) and 1.3 μL/mL of the β-tubulin antibody (1 mg/mL; Cat. MA1-19187, Thermo Fisher Scientific) per well and incubating for 1 h. After two washes with BB, we added 12.5 μL of the secondary staining solution in BB (1:500 of 1 mg/mL of GAM-DyLight 550; Cat. 84540, Thermo Fisher Scientific) and incubated for 1 h. The cells were then washed three times with WB and nuclei were stained with 0.1 μL/mL Hoechst 33342 (20 mM; Cat. 62249, Thermo Fisher Scientific).

Panel 2 (ER/lysosomes/membrane): Cells were incubated with stains for the ER (1 μL/mL of 1 mM ER-Tracker Blue-White DPX; Cat. E12353, Thermo Fisher Scientific) and lysosomes (0.2 μL/mL of 1 mM LysoTracker Red DND-99; Cat. L7528, Thermo Fisher Scientific) in pre-warmed DMEM for 30 min at 37 °C in a 5% CO_2_ atmosphere. After fixation as above, cells were washed twice with WB and incubated with 5 μL/mL labeled wheat germ agglutinin (1 mg/mL WGA Alexa Fluor 488 Conjugate; Cat. W11261, Thermo Fisher Scientific) for 10 min. WGA target intracellular membranes of the Golgi, lysosomes and nucleus [85,86] when incubated with cells for more than 1 h. However, our short incubation of 10 min avoids cellular uptake and staining is thus restricted to the plasma membrane, if undamaged.

Panel 3 (mitochondria/NF-κB): Cells were incubated with 0.17 μL/mL MitoTracker Orange CMTMRos (1 mM; Cat. M7510, Thermo Fisher Scientific) in DMEM for 30 min at 37 °C in a 5% CO_2_ atmosphere to stain thiol membranes [87]. After fixation as above, the cells were permeabilized, washed twice with WB, and incubated with the primary staining solution, including the antibody against NF-κB p65 (1 mg/mL; Cat. PA5-16545, Thermo Fisher Scientific) for 1 h. The primary antibody solution was removed and the cells were incubated in WBII for 15 min, washed twice with WB, and incubated with the secondary staining solution in WB (1:500 of 1 mg/mL of GAR-DyLight 550; Cat. 84541, Thermo Fisher Scientific) for 1 h. The cells were then incubated with WBII for 10 min and stained with 0.1 μL/mL Hoechst 33342 for another 10 min.

Panel 4 (p53/Caspase 9): After fixation as above, cells were permeabilized for 17 min, washed twice with WB, and blocked for 30 min. The BB was removed and the cells were incubated with the primary antibody in BB (5.5 μL/mL of 0.05 mg/mL p53 antibody and 1.5 μL/mL of undiluted caspase 9 antibody, both from Thermo Fisher Scientific, Cat. MA5-12557 and PA5-17913) for 1 h. After two washes with WBII and one with WB, the secondary staining solution in WB (1:500 of 1 mg/mL of GAM-DyLight 550 and 1:500 of 1 mg/mL of GAR DyLight 488; Cat. 35552, Thermo Fisher Scientific) was added for 1 h. The staining solution was removed and the cells were washed once with WBII and stained with Hoechst 33342 for 10 min.

Data analysis—general aspects. The screening assay involved nine cellular markers and a total of 115 cytological parameters (Table A3). The raw dataset was standardized (all data points were drawn onto a distribution centered at 1.0, which correspond to the controls). Furthermore, for simplicity, the image analysis data reported all features for all markers we measured. Therefore, the dataset was reduced according to biological relevance, manufacturer specifications for spot and non-spot measurements, and relevant literature [88,89], which indicate, for example, the importance of identifying and removing correlated features by scatterplot arrays, herein performed in STATISTICA v12.7 (Statsoft, Tulsa, OK, USA) [90]. The reduced dataset of 31 features was tested for normality and no normal distribution was found, so a non-parametric test was used for subsequent cluster analysis.

**Table A3:** Labeled cellular compartments, with additional information on markers and marker-target binding.

Target	Marker	Marker Binding Site (Data from Manufacturer, If Not Otherwise Cited)
**NF-κB**	NFkappaB/p65 antibody (PA5-16545, Thermo Fisher Scientific)	Binds to the activated subunit p65 of the heterodimer of NF-κB
**p53**	P53 antibody (MA5-12557, Thermo Fisher Scientific)	Stains the protein p53 in the nucleus and cytoplasma, but accumulates mainly in the nucleus. Process of subcellular localization still unclear.
**Caspase 9**	Cleaved caspase-9 antibody (PA5-17913, Thermo Fisher Scientific)	Stains the activated form of caspase 9 (cleaved caspase 9)
**Nucleus**	Hoechst 33342 (62249, Thermo Fisher Scientific)	Cell-permeable nucleic acid stain which emits blue fluorescence when bound to dsDNA
**Cell cycle**	(see nucleus)	
**Cell count**	(see nucleus)	
**Actin**	Phalloidin, Fluorescin Isothiocynate labeled (P5282, Sigma Aldrich)	Stains and stabilizes F-Actin
**Mitochondrion**	MitoTracker Orange CMTMros (M7510, Thermo Fisher Scientific)	The cell-permeant MitoTracker probes contain a mildly thiol-reactive compound. Thiols are redox-systems found in the mitochondrial matrix [87]
**Endoplasmic reitculum** **(ER)**	ER-Tracker Blue-White DPX (E-12353, Thermo Fisher Scientific)	Highly selective for ER but by unknown mechanism
**Lysosome**	Lysotracker Red DND-99 (L-7528, Thermo Fisher Scientific)	Stains acidic organelles in living cells
**Whole cell**	(see plasma membrane)	
**Plasma membrane**	Wheat germ agglutinin, Alexa Fluor 488 Conjugate (W11261, Thermo Fisher Scientific)	Wheat germ agglutinin selectively binds to peptidoglycans N-acetylglucosamine and N-acetylneuraminic acid (sialic acid) residues, found in the extracellular matrix.

Data analysis—definitions and detailed procedure. The HCS Cellomics manual (Thermo Fisher Scientific) provides the following definitions:

(a) Circ (C): “Circ is a cellular region derived from, and similar to, the area covered by the primary object; you can make the Circ larger or smaller than the primary object. The Circ is used to quantify the presence of a fluorescent macromolecule within the large cellular compartment defined by the primary object”. The primary object is very often, and also herein, the nucleus.
(b) Ring (R): “Ring is an annular region defined beyond the primary object. The position of the rings’ inner and outer perimeters can be defined in relation to the primary object’s location”. The ring region mostly covers the cytoplasm if the primary object is the nucleus.
(c) Ring Spots (R_Sp): “Ring Spots are any discrete punctate objects that fall within the Ring area. […] These spots can be used to identify discrete organelles or other punctate objects that are located in the cell’s cytoplasm. Examples of organelles that can be identified by this feature include: mitochondria, proteasomes, lysosomes, and endosomes”.
(d) Circ Spots (C_Sp): “Circ Spots are any discrete punctate objects that fall within the Circ area. Intensity thresholds identify these spots in a similar manner as for the Ring Spots. These spots can be organelles similar to those identified in Ring Spots, but in the cytoplasmic region above the nucleus, if the nucleus is the primary channel marker”. Not all cells are recorded on the same level, therefore C-Sp may include overlaying signals from the nucleus or cytoplasm (C-Sp).

As stated above, the number of parameters included in the evaluation of HCS data was reduced in stepwise manner. The abandoned parameters are described below along with the reasons for discarding them (nf = NF-κB; cp = caspase 9; wc = whole cell; Nuc = nucleus; ac = actin; mt = mitochondrion; er = endoplasmic reticulum; ly = lysosome; mb = plasma membrane).

(a) Biological redundancy: For example, the mitochondria were measured in the Circ and Ring regions, but the Circ region is focused on the nucleus so no representative mitochondrial signals are recorded here. Furthermore, caspase 9 is not translocated into the nucleus. Accordingly, we removed:
  mt_C_Ti	mt_C_SpAi	cp_C_Ti
  mt_C_AI	mt_C_SpTAr	cp_C_Ai
  mt_Rat-C-R	mt_C_SpAAr	cp_Rat-C-R
  mt_C_SpTi	mt_C_SpC	
(b) Spot vs. non-spot records: Based on the definition given above, we selected the spot records for discrete objects such as organelles, and non-spot measurements for continuous records such as actin, transcription factors and the membrane. Therefore, we removed:
  nf_R_SpTI	p53_C_SpTAr	ac_R_SpC
  nf_R_SpAI	p53_C_SpAAr	ac_C_SpTI
  nf_R_SpTAr	p53_C_SpC	ac_C_SpAI
  nf_R_SpAAr	cp_R_SpTI	ac_C_SpTAr
  nf_R_SpC	cp_R_SpAI	ac_C_SpAAr
  nf_C_SpTI	cp_R_SpTAr	ac_C_SpC
  nf_C_SpAI	cp_R_SpAAr	mb_R_SpTI
  nf_C_SpTAr	cp_R_SpC	mb_R_SpAI
  nf_C_SpAAr	cp_C_SpTI	mb_R_SpTAr
  nf_C_SpC	cp_C_SpAI	mb_R_SpAAr
  p53_R_SpTI	cp_C_SpTAr	mb_R_SpC
  p53_R_SpAI	cp_C_SpAAr	mb_C_SpTI
  p53_R_SpTAr	cp_C_SpC	mb_C_SpAI
  p53_R_SpAAr	ac_R_SpTI	mb_C_SpTAr
  p53_R_SpC	ac_R_SpAI	mb_C_SpAAr
  p53_C_SpTI	ac_R_SpTAr	mb_C_SpC
  p53_C_SpAI	ac_R_SpAAr	
(c) Select average records: Average records are normalized against the area of the cell (the definition from the Thermo Fisher Scientific manual is provided below). Given that swelling can occur when cells are exposed to stress factors such as envenomation, the total intensity could overestimate the individual records, hence the recommendation to use averages.

“For each cell, the primary object’s total intensity is measured. The total intensity is the sum of the intensities of all the individual pixels making up that object. The average intensity of the pixels making up the object is also reported.

$$\mathrm{Average}\;\mathrm{intensity}\;\mathrm{in}\;a\;\mathrm{region}=\frac{\mathrm{Total}\;\mathrm{intensity}\;\mathrm{in}\;\mathrm{the}\;\mathrm{region}}{\mathrm{Number}\;\mathrm{of}\;\mathrm{pixels}\;\mathrm{in}\;\mathrm{the}\;\mathrm{region}}$$

Therefore, we removed:

nf_R_TI	ac_C_TI	ly_C_TI
nf_C_TI	mt_R_TI	ly_C_SpTI
p53_R_TI	mt_R_SpTI	ly_C_SpTAr
p53_C_TI	mt_R_SpTAr	mb_R_TI
cp_R_TI	er_C_TI	mb_C_TI
Nuc_TI	er_C_SpTI	
ac_R_TI	er_C_SpTAr	

(d) Correlation: Some of the parameters contain the same information and thus appear to be correlated. Therefore, to avoid overestimating certain records, we removed one of both correlating parameters in the remaining dataset:
  wc_Size	correlated to wc_Area (R^2^ = 0.99)
  Nuc_Size	correlated to Nuc_Area (R^2^ = 0.97)
  ac_C_AI	correlated to ac_R_Ai (R^2^ = 0.99)
  er_C_AI	correlated to er_C_SpAi (R^2^ = 0.95)

The final HCS dataset includes the following 31 highly-informative parameters, with A to AF notations corresponding to those in Figure 2C in the main paper.

**A**	**nf_R-AI**	**I**	**wc_P2A**	**Q**	**ac_Rat-C-R**	**Z**	**ly_C_AI**
**B**	nf_C-AI	**J**	wc_LWR	**R**	mt_R-AI	**AA**	ly_C_SpAI
**C**	nf_Rat-C-R	**K**	Nuc_Area	**T**	mt_R-SpAI	**AB**	lyC_SpAAr
**D**	p53_R-AI	**L**	Nuc-P2A	**U**	mt_R-SpAAr	**AC**	ly_C_SpC
**E**	p53_C-AI	**M**	Nuc-LWR	**V**	mt_R-SpC	**AD**	mbR-AI
**F**	p53_Rat-C-R	**N**	Nuc-AI	**W**	er_C_SpAI	**AE**	mb_C-AI
**G**	cp_R-AI	**O**	Nuc-VI	**X**	er_C_SpAAr	**AF**	mb_Rat-C-R
**H**	wc_Area	**P**	ac_R-AI	**Y**	er_C_SpC		

## Appendix C. Additional Details on Cluster Identification

Pain cluster. The annotated functional categories of the transcriptome and high-content bioactivity screening datasets were compared, revealing that both datasets infer a subset of 29 pathways through which stingray toxins potentially exert their action. These pathways belong to 15 subclasses, the most abundant of which were *Signal transduction*, *Signaling molecules and interaction*, the *Circulatory system*, and the *Endocrine system* (Dataset S9). Each subclass contained potential new drug candidates.

Fish venoms are defensive weapons that have evolved primarily to cause pain, thus not surprisingly within the large functional data network we identified a cluster of transcriptome and bioactivity hits that predicts the MoA of pain-inducing stingray toxins. Pain is a complex process involving transduction, conduction, transmission, perception and modulation, throughout the nervous system. Furthermore, toxins are extracellular stimuli that induce signal transduction. Therefore, among the 29 shared pathways only those related to the *Nervous system*, *Sensory system*, *Signal Transduction* and *Signaling molecules and interaction* subclasses were specifically analyzed in the pain cluster (Dataset S9 ‘Pain Cluster’). These comprise nine pathways: *Dopaminergic synapse*, *Inflammatory mediator regulation of TRP channels*, *Calcium*-, *cAMP*-, *cGMP-PKG*-, *Apelin*-, *TGF-β*-, *TNF-* signaling pathways, and *Neuroactive ligand-receptor interaction*.

The functional subclasses are not solely targeted by pain-inducing toxins. Therefore, all transcriptome and bioactivity screening hits associated with these nine ‘pain’ pathways were manually filtered by their known relation to the pain system. Nine transcriptome hits were attributed, including five that were pain-related, four hemotoxins, and 11 against EF-hand proteins. The five pain-related hits comprised three venom nerve growth factors, endothelin, and cholecystoxin. The HCS contributed to these core pathways with 41 clustered reference drugs—predominantly GPCR-targeting neuropsychiatric, cardiovascular and anti-allergic drugs, such as fenoldopam bromide and ebastine. All of these drugs were included in the subsequent evaluation of the pain cluster because their molecular targets are involved in the activation, inhibition or modulation of pain signaling.

Cardiovascular and hemostasis cluster. Fish venoms also induce hypotensive crisis, blood pressure variations and injury-related hemorrhage [21]. We further identified clusters of putative toxins targeting the cardiovascular system and hemostasis. Similar tothe pain cluster, among the 29 shared pathways in the integrated dataset only those related to *Signal Transduction*, *Signaling molecules and interaction*, *Circulatory system*, and *Cardiovascular diseases* were considered for the cardiovascular cluster. These comprise 11 pathways: *Neuroactive ligand-receptor interaction*, *Dilated cardiomyopathy*, *Cardiac muscle contraction*, *Adrenergic signaling in cardiomyocytes*, *Vascular smooth muscle contraction* and signaling via the *Calcium-*, *cAMP-*, *cGMP-PKG-*, *Apelin-*, *TGF-β-* and *TNF* pathways. The *Hematopoietic cell lineage* pathway was added to gain insight into hemostasis-disrupting processes (Dataset S9).

As before, the selected functional subclasses were targeted by more than cardiovascular toxins. Therefore, all transcriptome and HCS hits associated with these 12 pathways were manually filtered by their known relationship with the cardiovascular and hemostasis systems. Thirty-six transcriptome hits were attributed, 17 of which were related to cardiovascular pathways, whereas nine were hemotoxins, and 11 were annotated EF-hand proteins. The putative cardiotoxins were bibrotoxin, cholecystoxin and 15 acidic/basic phospholipase A2 proteins. Additionally, we found a natriuretic peptide that was added due to clear cardiovascular relationship although expression level was below our threshold value, and thus needs further confirmation (Figure A2).

The putative hemotoxins included venom plasminogen activator, thrombin-like enzyme, turripeptide, snaclec A8, C-type lectin lectoxin, and four additional PLA2 proteins with potential hemotoxic activity. The HCS contributed to this cluster with 41 clustered reference drugs, among which 14 have a cardiovascular indication evident from their annotated drug class and activity. These include amiodarone HCl, bretylium tosylate, and phenoxybenzamine HCl. The functional data representing these compounds were subsequently used to provide initial insight into the mechanism of action of putative cardiovascular and hemotoxins from the target to the cellular response (Figure A2 and Figure A3).

## Appendix D. Phospholipase A2 and Putative Three-Finger Toxins

Multiple phospholipases A2 (PLA2) candidates were identified in the five newly sequenced and three reanalyzed transcriptomes of stingray venomous tissues. These were aligned to active Asp(D)49-PLA2 and inactive Lys(K)49-PLA2 (Figure A4) representing two PLA2 subgroups that differ mainly in the residue found at position 49 (aspartate defining the active subfamily and lysine the inactive subfamily) [91]. In all sequences we analyzed, an aspartate was found at position 49, indicating that all hits represented the active D49 subfamily (Figure A4). Given the absence of PLA2 activity in the stingray venoms analyzed thus far [26] it is possible that the candidates are non-venomous PLA2 proteins from the epidermal spine tissue, a topic that requires further analysis and clarification.

We also identified several members of the three-finger toxin (TFT) family, which has thus far been found only in snake venoms [92]. The stingray homologs showed particular similarity to TFT proteins from sea snakes. We aligned the stingray transcripts to known TFTs from proteomic and transcriptomic studies to determine whether the typical cysteine scaffold attributed to this family was present (Figure A5). The TFT family also includes several non-toxic proteins containing three-finger domains, but unlike the genuine toxins these possess a C-terminal glycosylphosphatidylinositol (GPI) anchor. We therefore revised our candidate TFT transcripts by using GPI-SOM [93], PredGPI [94], bigGPI [95], and Net-GPI [96] to predict the presence or absence of a GPI anchor. We found that a three finger cysteine scaffold was present in most of the transcripts encoding proteins with >90% sequence similarity to known TFTs, whereas those with 40–50% similarity contained a less congruent cysteine scaffold, probably reflecting sequence truncation or false-positive annotations. Three of 50 transcripts in our eight transcriptomes were clearly predicted to encode with GPI anchors (and a valid signal peptide prediction), all of which were among those 40–50% similarity to known TFTs. These results indicate that some of our hits (especially those with low sequence similarity) may represent non-toxic three-finger proteins rather than genuine TFTs, and may also represent inactive homologs lacking a complete cysteine scaffold, which may also indicate incorrect assignment to the TFT family. However, the 10 transcripts encoding proteins with >90% sequence similarity to sea snake TFT proteins contained a correct cysteine scaffold and were predicted to lack a GPI anchor, supporting the presence of TFT-like proteins in stingray venomous tissue.

## References

[1] Jenner R.A., Undheim E. (2017). Venom: The Secrets of Nature’s Deadliest Weapon.

[2] Holford M., Daly M., King G.F., Norton R.S. (2018). Venoms to the rescue. Science.

[3] Arbuckle K., Gopalakrishnakone P., Malhotra A. (2015). Evolutionary context of venom in animals. Evolution of Venomous Animals and Their Toxins.

[4] Dutertre S., Jin A.H., Vetter I., Hamilton B., Sunagar K., Lavergne V., Dutertre V., Fry B.G., Antunes A., Venter D.J. (2014). Evolution of separate predation-and defence-evoked venoms in carnivorous cone snails. Nat. Comm..

[5] Pineda S.S., Chin Y.K.Y., Undheim E.A., Senff S., Mobli M., Dauly C., Escoubas P., Nicholson G.M., Kaas Q., Guo S. (2020). Structural venomics reveals evolution of a complex venom by duplication and diversification of an ancient peptide-encoding gene. Proc. Natl. Acad. Sci. USA.

[6] Mebs D. (2002). Venomous and Poisonous Animals: A Handbook for Biologists, Toxicologists and Toxinologists, Physicians and Pharmacists.

[7] Davis J., Jones A., Lewis R.J. (2009). Remarkable inter-and intra-species complexity of conotoxins revealed by LC/MS. Peptides.

[8] Casewell N.R., Wüster W., Vonk F.J., Harrison R.A., Fry B.G. (2013). Complex cocktails: The evolutionary novelty of venoms. Trends Ecol. Evol..

[9] FDA Drugs@FDA; Search Capoten®. 6 April 1981. https://www.accessdata.fda.gov/scripts/cder/daf/index.cfm?event=overview.process&ApplNo=018343.

[10] Takacs Z., Nathan S., Wexler P. (2014). Animal venoms in medicine. Encyclopedia of Toxicology.

[11] Fusetani N., Kem W. (2009). Marine Toxins as Research Tools.

[12] Debono J., Xie B., Violette A., Fourmy R., Jaeger M., Fry B.G. (2017). Viper venom botox: The molecular origin and evolution of the waglerin peptides used in anti-wrinkle skin cream. J. Mol. Evol..

[13] Escoubas P., King G.F. (2009). Venomics as a drug discovery platform. Expert Rev. Proteom..

[14] Hutter B., Schaab C., Albrecht S., Borgmann M., Brunner N.A., Freiberg C., Ziegelbauer K., Rock C.O., Ivanov I., Loferer H. (2004). Prediction of mechanisms of action of antibacterial compounds by gene expression profiling. Antimicrob. Agents Chemother..

[15] Hay M., Thomas D.W., Craighead J.L., Economides C., Rosenthal J. (2014). Clinical development success rates for investigational drugs. Nat. Biotechnol..

[16] Silva A., Isbister G.K. (2020). Current research into snake antivenoms, their mechanisms of action and applications. Biochem. Soc. Trans..

[17] Yıldırım M.A., Goh K.I., Cusick M.E., Barabási A.L., Vidal M. (2007). Drug—Target network. Nat. Biotechnol..

[18] Hopkins A.L. (2008). Network pharmacology: The next paradigm in drug discovery. Nat. Chem. Biol..

[19] Casas A.I., Hassan A.A., Larsen S.J., Gomez-Rangel V., Elbatreek M., Kleikers P.W., Funey E., Egea J., Lopez M.G., Baumbach J. (2019). From single drug targets to synergistic network pharmacology in ischemic stroke. Proc. Natl. Acad. Sci. USA.

[20] Boezio B., Audouze K., Ducrot P., Taboureau O. (2017). Network-based approaches in pharmacology. Mol. Inform..

[21] Ziegman R., Alewood P. (2015). Bioactive components in fish venoms. Toxins.

[22] Jared C., Mailho-Fontana P.L., Antoniazzi M.M., Mendes V.A., Barbaro K.C., Rodrigues M.T., Brodie E.D. (2015). Venomous frogs use heads as weapons. Curr. Biol..

[23] Von Reumont B.M., Campbell L.I., Jenner R.A. (2014). Quo vadis venomics? A roadmap to neglected venomous invertebrates. Toxins.

[24] Smith W.L., Wheeler W.C. (2006). Venom evolution widespread in fishes: A phylogenetic road map for the bioprospecting of piscine venoms. J. Hered..

[25] Harris R.J., Jenner R.A. (2019). Evolutionary ecology of fish venom: Adaptations and consequences of evolving a venom system. Toxins.

[26] Barbaro K.C., Lira M.S., Malta M.B., Soares S.L., Neto D.G., Cardoso J.L., Santoro M.L., Haddad V. (2007). Comparative study on extracts from the tissue covering the stingers of freshwater (*Potamotrygon falkneri*) and marine (*Dasyatis guttata*) stingrays. Toxicon.

[27] Pedroso C.M., Jared C., Charvet-Almeida P., Almeida M.P., Neto D.G., Lira M.S., Haddad V., Barbaro K.C., Antoniazzi M.M. (2007). Morphological characterization of the venom secretory epidermal cells in the stinger of marine and freshwater stingrays. Toxicon.

[28] Froese R., Pauly D. FishBase. WorldWideWeb Electronic Publication. Version 6. www.fishbase.org.

[29] De Oliveira N.G., da Rocha Fernandes G., Cardoso M.H., Costa F.F., de Souza Cândido E., Neto D.G., Mortari M.R., Schwartz E.F., Franco O.L., De Alencar S.A. (2016). Venom gland transcriptome analyses of two freshwater stingrays (Myliobatiformes: Potamotrygonidae) from Brazil. Sci. Rep..

[30] Silva F., Huang Y., Yang V., Mu X., Shi Q., Antunes A. (2018). Transcriptomic characterization of the South American freshwater stingray *Potamotrygon motoro* venom apparatus. Toxins.

[31] Hefti F.F., Rosenthal A., Walicke P.A., Wyatt S., Vergara G., Shelton D.L., Davies A.M. (2006). Novel class of pain drugs based on antagonism of NGF. Trends Pharmacol. Sci..

[32] Berridge M.J., Lipp P., Bootman M.D. (2000). The versatility and universality of calcium signalling. Nat. Rev. Mol. Cell Biol..

[33] Raffaello A., Mammucari C., Gherardi G., Rizzuto R. (2016). Calcium at the center of cell signaling: Interplay between endoplasmic reticulum, mitochondria, and lysosomes. Trends Biochem. Sci..

[34] Haddad V., Neto D.G., de Paula Neto J.B., de Luna Marques F.P., Barbaro K.C. (2004). Freshwater stingrays: Study of epidemiologic, clinic and therapeutic aspects based on 84 envenomings in humans and some enzymatic activities of the venom. Toxicon.

[35] Conceição K., Konno K., Melo R.L., Marques E.E., Hiruma-Lima C.A., Lima C., Richardson M., Pimenta D.C., Lopes-Ferreira M. (2006). Orpotrin: A novel vasoconstrictor peptide from the venom of the Brazilian stingray *Potamotrygon gr. orbignyi*. Peptides.

[36] Baumann K., Casewell N.R., Ali S.A., Jackson T.N., Vetter I., Dobson J.S., Cutmore S.C., Nouwens A., Lavergne V., Fry B.G. (2014). A ray of venom: Combined proteomic and transcriptomic investigation of fish venom composition using barb tissue from the blue-spotted stingray (*Neotrygon kuhlii*). J. Proteom..

[37] Magalhaes K.W., Lima C., Piran-Soares A.A., Marques E.E., Hiruma-Lima C.A., Lopes-Ferreira M. (2006). Biological and bio-chemical properties properties of the Brazilian *Potamotrygon* stingrays: *Potamotrygon cf. scobina* and *Potamotrygon gr. orbignyi*. Toxicon.

[38] Sade Y.B., Bóia-Ferreira M., Gremski L.H., da Silveira R.B., Gremski W., Senff-Ribeiro A., Chaim O.M., Veiga S.S. (2012). Molecular cloning, heterologous expression and functional characterization of a novel translationally-controlled tumor protein (TCTP) family member from *Loxosceles intermedia* (brown spider) venom. Int. J. Biochem. Cell Biol..

[39] Magalhães M.R., da Silva N.J., Ulhoa C.J. (2008). A hyaluronidase from *Potamotrygon motoro* (freshwater stingrays) venom: Isolation and characterization. Toxicon.

[40] Kumar K.R., Vennila R., Kanchana S., Arumugam M., Balasubramaniam T. (2011). Fibrinogenolytic and anticoagulant activities in the tissue covering the stingers of marine stingrays *Dasyatis sephen* and *Aetobatis narinari*. J. Thromb. Thrombolysis.

[41] Gutiérrez J.M., Lomonte B. (2013). Phospholipases A2: Unveiling the secrets of a functionally versatile group of snake venom toxins. Toxicon.

[42] De LM Junqueira-de-Azevedo I., Pertinhez T., Spisni A., Carreño F.R., Farah C.S., Ho P.L. (2003). Cloning and expression of calglandulin, a new EF-hand protein from the venom glands of *Bothrops insularis* snake in *E. coli*. Biochim. Biophys. Acta Proteins Proteomics.

[43] Liu Y., Beyer A., Aebersold R. (2016). Aebersold, On the dependency of cellular protein levels on mRNA abundance. Cell.

[44] Lee C.C., Hsieh H.J., Hsieh C.H., Hwang D.F. (2015). Plancitoxin I from the venom of crown-of-thorns starfish (*Acanthaster planci*) induces oxidative and endoplasmic reticulum stress associated cytotoxicity in A375.S2 cells. Exp. Mol. Pathol..

[45] Rajeshkumar R.K., Vennila R., Karthikeyan S., Prasad N.R., Arumugam M., Velpandian T., Balasubramaniam T. (2015). Anti-proliferative activity of marine stingray *Dasyatis sephen* venom on human cervical carcinoma cell line. J. Venom. Anim. Toxins. Incl. Trop. Dis..

[46] Moreira L., Gutiérrez J., Borkow G., Ovadia M. (1992). Ultrastructural alterations in mouse capillary blood vessels after experimental injection of venom from the snake *Bothrops asper* (terciopelo). Exp. Mol. Pathol..

[47] Feofanov A.V., Sharonov G.V., Astapova M.V., Rodionov D.I., Utkin Y.N., Arseniev A.S. (2005). Cancer cell injury by cytotoxins from cobra venom is mediated through lysosomal damage. Biochem. J..

[48] Ebrahim K., Shirazi F.H., Mirakabadi A.Z., Vatanpour H. (2015). Cobra venom cytotoxins; apoptotic or necrotic agents?. Toxicon.

[49] Jouiaei M., Yanagihara A.A., Madio B., Nevalainen T.J., Alewood P.F., Fry B.G. (2015). Ancient venom systems: A review on cnidaria toxins. Toxins.

[50] Swinney D.C., Anthony J. (2011). How were new medicines discovered?. Nat. Rev. Drug Discov..

[51] Schenone M., Dančík V., Wagner B.K., Clemons P.A. (2013). Target identification and mechanism of action in chemical biology and drug discovery. Nat. Chem. Biol..

[52] Young D.W., Bender A., Hoyt J., McWhinnie E., Chirn G.W., Tao C.Y., Tallarico J.A., Labow M., Jenkins J.L., Mitchison T.J. (2008). Integrating high-content screening and ligand-target prediction to identify mechanism of action. Nat. Chem. Biol..

[53] Trim S.A., Trim C.M. (2013). Venom: The sharp end of pain therapeutics. Br. J. Pain.

[54] Daniel J.T., Clark R.J. (2017). G-protein coupled receptors targeted by analgesic venom peptides. Toxins.

[55] Cuypers E., Peigneur S., Debaveye S., Shiomi K., Tytgat J. (2012). TRPV1 channels as new targers for marine toxins: Example of gigantoxin I, a sea anemone toxin acting via modulation of the PLA2 pathway. Acta Chim. Slov..

[56] Yazawa K., Wang J.W., Hao L.Y., Onoue Y., Kameyama M. (2007). Verrucotoxin, a stonefish venom, modulates calcium channel activity in guinea-pig ventricular myocytes. Br. J. Pain.

[57] Snutch T.P. (2005). Targeting chronic and neuropathic pain: The N-type calcium channel comes of age. NeuroRx.

[58] Callaghan B., Haythornthwaite A., Berecki G., Clark R.J., Craik D.J., Adams D.J. (2008). Analgesic α-conotoxins Vc1. 1 and Rg1A inhibit N-type calcium channels in rat sensory neurons via GABAB receptor activation. J. Neurosci..

[59] Pinho-Ribeiro F.A., Verri W.A., Chiu I.M. (2017). Nociceptor sensory neuron–immune interactions in pain and inflammation. Trends Immunol..

[60] Weisel-Eichler A., Libersat F. (2004). Venom effects on monoaminergic systems. J. Comp. Physiol..

[61] Russell F.E. (1965). Marine toxins and venomous and poisonous marine animals. Advances in Marine Biology.

[62] Barr T.P., Kam S., Khodorova A., Montmayeur J.P., Strichartz G.R. (2011). New perspectives on the endothelin axis in pain. Pharmacol. Res..

[63] Lovick T.A. (2008). Pro-nociceptive action of cholecystokinin in the periaqueductal grey: A role in neuropathic and anxiety-induced hyperalgesic states. Neurosci. Biobehav. Rev..

[64] Grabherr M.G., Haas B.J., Yassour M., Levin J.Z., Thompson D.A., Amit I., Adiconis X., Fan L., Raychowdhury R., Zeng Q. (2011). Trinity: Reconstructing a full-length transcriptome without a genome from RNA-Seq data. Nat. Biotechnol..

[65] Bushmanova E., Antipov D., Lapidus A., Prjibelski A.D. (2019). rnaSPAdes: A de novo transcriptome assembler and its application to RNA-Seq data. GigaScience.

[66] Kim D., Langmead B., Salzberg S.L. (2015). HISAT: A fast spliced aligner with low memory requirements. Nat. Methods.

[67] Altschul S.F., Gish W., Miller W., Myers E.W., Lipman D.J. (1990). Basic local alignment search tool. J. Mol. Biol..

[68] Eddy S.R. (2009). A new generation of homology search tools based on probabilistic inference. Genome Inform..

[69] UniProt Consortium (2019). UniProt: A worldwide hub of protein knowledge. Nucleic Acids Res..

[70] El-Gebali S., Mistry J., Bateman A., Eddy S.R., Luciani A., Potter S.C., Qureshi M., Richardson L.J., Salazar G.A., Smart A. (2019). The Pfam protein families database in 2019. Nucleic Acids Res..

[71] Jungo F., Bougueleret L., Xenarios I., Poux S. (2012). The UniProtKB/Swiss-Prot Tox-Prot program: A central hub of inte-grated venom protein data. Toxicon.

[72] Kanehisa M., Goto S. (2000). KEGG: Kyoto encyclopedia of genes and genomes. Nucleic Acids Res..

[73] Kanehisa M., Furumichi M., Tanabe M., Sato Y., Morishima K. (2017). KEGG: New perspectives on genomes, pathways, diseases and drugs. Nucleic Acids Res..

[74] Gustafsdottir S.M., Ljosa V., Sokolnicki K.L., Wilson J.A., Walpita D., Kemp M.M., Seiler K.P., Carrel H.A., Golub T.R., Schreiber S.L. (2013). Multiplex cytological profiling assay to measure diverse cellular states. PLoS ONE.

[75] Saeed A., Sharov V., White J., Li J., Liang W., Bhagabati N., Braisted J., Klapa M., Currier T., Thiagarajan M. (2003). TM4: A free, open-source system for microarray data management and analysis. BioTechniques.

[76] Schulze C.J., Bray W.M., Woerhmann M.H., Stuart J., Lokey R.S., Linington R.G. (2013). “Function-first” lead discovery: Mode of action profiling of natural product libraries using image-based screening. Chem. Biol..

[77] Andrews S. FASTQC. A Quality Control Tool for High Throughput Sequence Data. http://www.bioinformatics.babraham.ac.uk/projects/fastqc/.

[78] Martin M. (2011). Cutadapt removes adapter sequences from high-throughput sequencing reads. EMBnet J..

[79] Schmieder R., Edwards R. (2011). Quality control and preprocessing of metagenomic datasets. Bioinformatics.

[80] Brian H., Papanicolaou A. Transdecoder (Find Coding Regions Within Transcripts). http://transdecoder.github.io.

[81] Camacho C., Coulouris G., Avagyan V., Ma N., Papadopoulos J., Bealer K., Madden T.L. (2009). BLAST+: Architecture and applications. BMC Bioinform..

[82] Eddy S.R. (2011). Accelerated Profile HMM Searches. PLoS Comput. Biol..

[83] Pertea G.M., Antonescu C.M., Chang T.C., Mendell J.T., Salzberg S.L. (2015). StringTie enables improved reconstruction of a transcriptome from RNA-seq reads. Nat. Biotech..

[84] Kremb S., Voolstra C.R. (2017). High-resolution phenotypic profiling of natural products-induced effects on the single-cell level. Sci. Rep..

[85] Gabor F., Bogner E., Weissenboeck A., Wirth M. (2004). The lectin–cell interaction and its implications to intestinal lectin-mediated drug delivery. Adv. Drug Deliv. Rev..

[86] Bird-Lieberman E.L., Neves A.A., Lao-Sirieix P., O’donovan M., Novelli M., Lovat L.B., Eng W.S., Mahal L.K., Brindle K.M., Fitzgerald R.C. (2012). Molecular imaging using fluorescent lectins permits rapid endoscopic identification of dysplasia in Barrett’s esophagus. Nat. Med..

[87] Murphy M.P. (2012). Mitochondrial thiols in antioxidant protection and redox signaling: Distinct roles for glutathionylation and other thiol modifications. Antioxid. Redox Signal..

[88] Feng Y., Mitchison T.J., Bender A., Young D.W., Tallarico J.A. (2009). Multi-parameter phenotypic profiling: Using cellular effects to characterize small-molecule compounds. Nat. Rev. Drug Discov..

[89] Haney S.A., Haney S.A., Bowman D., Chakravarty A., Davies A., Shamu C. (2015). Factoring and clustering high content data. An Introduction to High Content Screening: Imaging Technology, Assay Development, and Data Analysis in Biology and Drug Discovery.

[90] Hill T., Lewicki P. (2007). STATISTICS: Methods and Applications.

[91] Jia Y., Ermolinsky B., Garza A., Provenzano D. (2017). Phospholipase A2 in the venom of three cottonmouth snakes. Toxicon.

[92] Utkin Y.N. (2019). Last decade update for three-finger toxins: Newly emerging structures and biological activities. World J. Biol. Chem..

[93] Fankhauser N., Maeser P. (2005). Identification of GPI-anchor signals by a Kohonen Self Organizing Map. Bioinformatics.

[94] Pierleoni A., Martelli P.L., Casadio R. (2008). PredGPI: A GPI anchor predictor. BMC Bioinform..

[95] Eisenhaber B., Bork P., Eisenhaber F. (1999). Prediction of potential GPI-modification sites in proprotein sequences. J. Mol. Biol..

[96] Gíslason M.H., Nielsen H., Armenteros J.J.A., Johansen A.R. (2019). Prediction of GPI-Anchored proteins with pointer neural networks. bioRxiv.

